# Childbirth Experience Questionnaire: Cross-cultural validation and psychometric evaluation for European Portuguese

**DOI:** 10.1177/17455057221128121

**Published:** 2022-10-18

**Authors:** Maria João Pimenta Marques, Otília Zangão, Luis Miranda, Margarida Sim-Sim

**Affiliations:** 1 Women’s and Children’s Department, Hospital do Espírito Santo de Évora (HESE-EPE), Évora, Portugal; 2 Comprehensive Health Research Centre (CHRC), University of Évora, Évora, Portugal; 3 Nursing Department, University of Évora, Évora, Portugal; 4 Centro Hospitalar Barreiro Montijo (CHBM), Hospital do Barreiro, Barreiro, Portugal

**Keywords:** labour obstetrics, midwifery, psychometrics, validation study, women

## Abstract

**Background:**

Self-reported measures are relevant both for the clinic and for health evaluation because they provide an interpretation of quality parameters. Women who experience labour can express themselves through these measures, identifying indicators that need improvement.

**Objective:**

The objective of this study is to adapt the Childbirth Experience Questionnaire to the Portuguese context and to determine its psychometric properties.

**Method:**

A methodological study carried out with a convenience sample where the participants were 161 female users of a hospital in southern Portugal. They were aged between 20 and 43 years (M = 31.05, SD = 4.87) and answered a questionnaire approximately 48 h postpartum, preserving the ethical principles. The original instrument, with 22 items, underwent the linguistic and cultural adequacy process.

**Results:**

Factor analysis with Varimax rotation was performed, revealing a set of 19 items with factor weights above .400. The set of items remained four-dimensional as the original, explaining 62.517% of the variance. In the retest, the reliability results showed that similar characteristics to the original study are maintained in the two subscales that express ‘Participation’ (three items) and ‘Professional Support’ (four items), with internal consistency values of .807 and .782. The ‘Own Performance’ and ‘Own Threshold’ subscales were elaborated from the results of the Varimax rotation, presenting Cronbach’s alpha coefficients of .840 and 714, respectively. The total scale showed alpha values of .873 and .823 in the test and retest, respectively. Time stability showed a positive association, with r = .659 (p < .001). Accuracy through the split-half method reached an alpha value of .880 with Spearman–Brown correction. The floor effect was high in the ‘Participation’ subscale, both in the test and in the retest. Convergent validity between the instrument and the ‘Index of Strategies for Pain Relief in Labour’ discrete variable showed a Spearman’s rho value of .209 (p = .011) in the total scale. In discriminating validity, the Mann–Whitney test reveals that the women who recognize interactions with the midwife have more favourable scores in Childbirth Experience Questionnaire (U = 2748.000; Z = 2.905; p = .004).

**Conclusion:**

The current version in European Portuguese suggests that it is a valid and reliable measure. This study may facilitate other validation processes in Lusophony countries.

## Introduction

Labour (LB) is an expression of continuity of the species, resulting from complex physiological processes. It is a unique and unrepeatable experience that leaves memories.

The reason for LB onset is not absolutely known and is explained by theories of changes in the oestrogen/progesterone ratio, by an increase in prostaglandin production, by progressive endometrial sensitivity to endogenous oxytocin, by stimulation of the Ferguson reflex or by the interaction between these factors, which occur in the maternal–foetal unit.^1,2^ A person who experienced the phenomenon in the role of parturient recounts the experience, from an ethnocentric perspective, recording the symptoms, progress, conditions of the surroundings and how she was cared for.^3,4^ In LB, the woman’s performance, the confrontation with her pain/discomfort thresholds, the request for professional support and the ability to participate actively, claiming her right to be the central figure of the event, entail positive experiences.^5,6^ However, negative experiences or those who fail to meet the expectations increase the risk of postpartum depression and even influence breastfeeding and family planning decisions, among others.^7^

Some of the situations that generate positive/negative experiences in parturient women are under the caregivers’ performance area, particularly midwives who manage the ‘birth territory’.^7–9^ These professionals work with specific knowledge, use instruments, methods and communication means, following institutional guidelines, international recommendations (e.g. World Health Organization; National Health System; International Council of Midwives) and their own care styles, influenced by the culture of the local professional community. This is how medicalized or naturalistic models are generated.^10^

Research on LB topics, either qualitatively or quantitatively, develops knowledge, and it is essential to have Patient-Reported Outcome Measures (PROMs) available.^11^ Such measures collect perspectives and allow evaluating experiences, making comparisons and recording evolution/regression. This can lead to efforts to improve care or not, this being the midwives’ mission, together with the women’s mother.^12^

The Childbirth Experience Questionnaire (CEQ)^5^ is a PROM that assesses women’s perception in relation to the care experience during delivery. Originally developed in Sweden, Northern Europe,^5^ CEQ was applied in women of several nationalities, both in Western Europe countries, such as the United Kingdom,^13^ and in Mediterranean Europe ones, such as Spain,^14^ in Central Europe (Slovakia)^15^ and even in Asia, namely in China,^16^ suggesting its usefulness in evaluating the phenomenon due to the diversity of languages and care contexts.

Despite cultural diversity, the LB experience as an ancestral phenomenon has similarities in the care processes, which are reflected in the parturient women’s experiences, who recount them from their perceptions and memories.^8,17,18^ Considering the already validated versions of CEQ, the participants have been 2340 puerperal women (206 English, 226 Spanish, 1747 Chinese and 161 Slovaks). In relation to the properties of the instrument, except in the Slovakian study where it was not evaluated,^15^ Cronbach’s alpha coefficient of the overall CEQ scale varied between .900^5^ and .880,^14,16^ showing good internal consistency. In the several languages, organization of the items in the subscales reveals dimensions that are not absolutely coincident^14,16^ but very close to the original scale. However, face validity^13^ is recognized in the validation procedures performed, and ease of use and time stability.^13^ The studies cited are unanimous in acknowledging CEQ as a valid and reliable measure of women’s perceptions.^13,14,16^ These studies suggest that its application can be a factor that promotes women’s empowerment during delivery^15^ and for care quality,^15,16^ and converging for positive childbirth experiences^19^

Women’s perception regarding their delivery experience is a phenomenon of interest in countries with low birth rates, such as Portugal, where there is no generational replacement since 1981 (Total Fertility Rate (TFR) = 2.13), dating from the 1980s, with a similar panorama in Slovakia (TFR = 2.14 in 1987), Spain (TFR = 2.04 in 1981) and the United Kingdom (TFR = 2.04 in 1973).^20^ It is important for health professionals to gather information from a PROM perspective, particularly in regions with scarce human resources in health and where the professionals/parturient women ratio presents gaps, cases in which there are almost no alternatives for non-medicalized deliveries.^21^ In fact, it is not uncommon that changes are achieved in care management through the users’ dissatisfaction. Some instruments that assess women’s perceptions regarding care are validated for Portuguese,^22^ although they were not specifically devised for parturient women, that is the reason why the evaluation is generalist in nature. In the Portuguese context and in the face of legal guidelines (Law No. 110/2019 dated 9 September) that recognize the need to assess women’s satisfaction regarding the care received, validation of the CEQ instrument can be useful. Actually, the constant rights set forth in articles 15-A and 15-F from Law No. 110/2019 include content close to the CEQ items. Therefore, there is a suggested urgent need for instruments validated in Portuguese in the research and care quality scope.

To perform such evaluation, the measures need to show adequate properties, which, in addition to linguistic–conceptual understanding, present adequate psychometric properties.

Having failed to identify, as far as it was possible to research, any version of the CEQ^5^ in European Portuguese, there is an evident gap, although recognizing advantages regarding availability of the instrument in the scope of care quality and good practices during the pregnancy–puerperal period^23^ for local studies and for multicentre ones.

## Objective

The objective of this study is to adapt the CEQ to the Portuguese context and to determine its psychometric properties.

## Method

This study is a methodological survey conducted by means of a cross-sectional approach.

### Participants

Convenience sample that invited 180 women, who were users of wards for puerperal women at a hospital in southern Portugal in 2019.

The inclusion criteria considered the following: (a) age equal to or greater than 18 years; (b) ability to read and write Portuguese; (c) pregnancy monitored in health services; (d) pregnancy lasting between 37 and 42 weeks, without complications and (e) vaginal delivery approximately 48 h ago. The exclusion criteria were as follows: (a) multiple pregnancy; (b) puerperal women with newborns manifesting health problems and (c) puerperal women with manifestations of mental health disorders.

## Data collection instrument

The questionnaire was organized into four sections. The first section corresponded to the sociodemographic data (i.e. age, marital status and educational qualifications), data from the obstetric history and LB (i.e. number of prenatal visits, pregnancy surveillance locus, duration of pregnancy, presentation of delivery plan at the date of admission to the maternity ward, type of delivery, epidural analgesia, and number of children). In the second section, questions about LB were asked through the following: (a) interval variable from 0 to 100, on ‘the level of support provided by the nurses for pain relief during LB’; (b) interval variable from 0 to 100, on ‘the level of pain intensity during LB’; (c) ‘Index of Strategies for Pain Relief in LB’ (ISPRL) and (d) the categorical variable (yes/no) called ‘Did the nurse ask several times during LB if you were OK?’.

The third section contained the CEQ scale. The last question requested contact for the implementation of CEQ at the second moment.

## ISPRL

**Figure 1.**
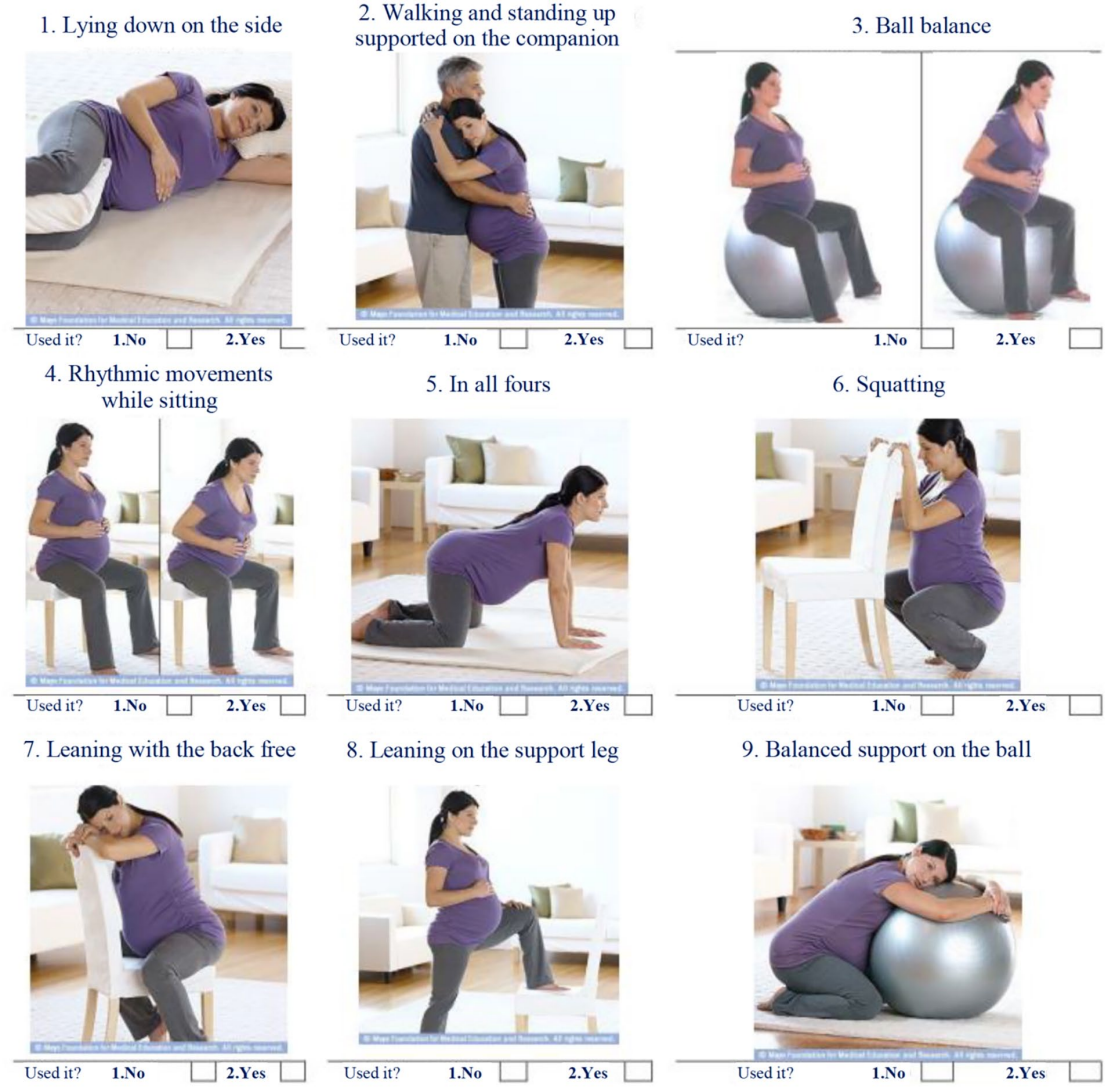
Questionnaire from the ISPRL.

The variable is presented through nine figures, with positions to be used for pain relief in LB. It is presented as dichotomous answers (*I used it* = 1; *I did not use it* = 0), from which the use sum was constituted. Permission was requested to the Mayo Clinic to use the figures,^24^ obtaining a positive response. Figure 1 illustrates the scheme presented to the participants.

## CEQ

CEQ is a latent variable that measures the mother’s perception of childbirth, formulating this experience through 22 manifest variables or items, such as ‘The labour progress went as I had expected’. Overall, 19 items are presented on a Likert-type scale, scored from 4 (*Totally disagree*) to 1 (*Totally agree*). The items with a positive formulation are reverted (i.e. 1, 2, 4, 6, 7, 10, 11, 12, 13, 14, 15, 16, 17, 18, 19). The highest score corresponds to the most satisfactory delivery experience. The remaining three items (20, 21 and 22) are presented on a visual scale from 0 to 100 points in a straight 100 mm line on the paper, on which the respondent points an X. Space in millimetres is measured and categorized according to the following intervals: 0–40 = 1, 41–60 = 2, 61–80 = 3 and 81–100 = 4. The original scale has four dimensions: (1) Own capacity dimension, concerning eight items (1, 2, 4, 5, 6, 19, 20 and 21); (2) Professional support dimension with five items (13, 14, 15, 16 and 17); (3) Perceived safety dimension with six items (3, 7, 8, 9, 18 and 22) and (4) Participation dimension with three items (10, 11 and 12), with Cronbach’s alpha coefficients of .82, .88, .78 and .62, respectively.^5^

## Intercultural equivalence

**Figure 2.**
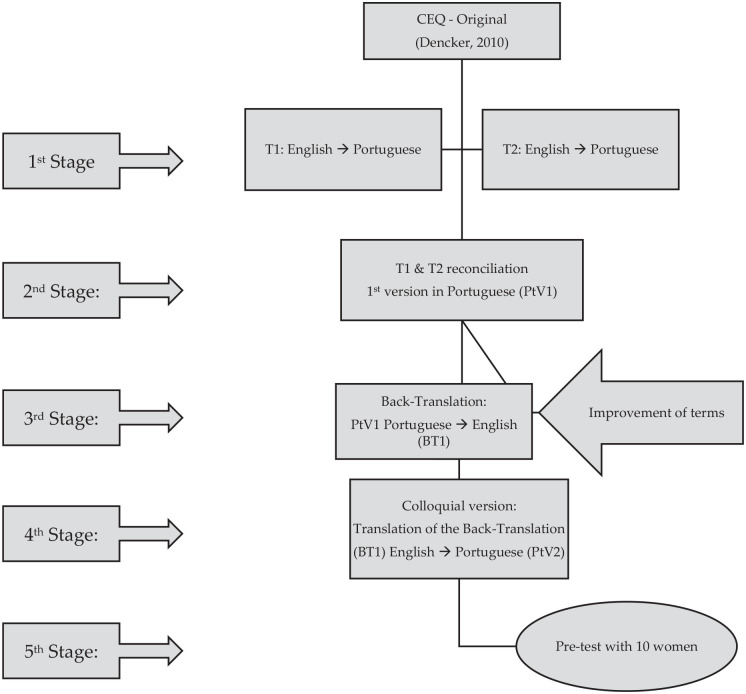
Stages of the cross-cultural adaptation.

The process was initiated from the original version published in English^5^ and followed a number of stages.^12^ In the first stage, the English–Portuguese translation was performed by a Portuguese health technician (T1), who is fluent in English and who worked in the United States and, separately, by a Portuguese nurse (T2), who is proficient in the language. In the second stage, one professional translator reconciled both Portuguese versions, creating the first version in Portuguese (PtV1). In the third stage, a bilingual American nursing professor performed the back-translation (BT1), verifying with one of the authors the differences in some terms, such as ‘LB *progressed* as I expected’ *versus* ‘The LB progress *went* as I expected’. In the fourth stage, a Health Science professor translated the instrument (BT1) into Portuguese (PtV2) to obtain a colloquial version, which was submitted to the authors with agreement in all the items. In the fifth stage, the Portuguese version was applied to 10 puerperal women, who orally confirmed understanding of the manifest variables, not suggesting changes in their formulation. The process is described in Figure 2.

## Sample size calculation

Sample size definition is a controversial aspect in validation studies. In some references, the number of participants depends on the characteristics of the instrument applied and should be around 300 subjects^12^ or between 200 and 300.^25,26^ Other suggestions characterize samples comprised by 100 subjects as deficient, 200 subjects as acceptable and 300 as good.^12^ A frequently applied rule is to consider a proportion of 5 or 10 subjects for each of the scale items.^12^ In this study, a proportion of 5 to 10 cases per item was considered, seeking to achieve a sample comprised by between 110 (22 items × 5 cases = 110) and 220 (22 items × 10 cases = 220). By agreeing upon a mid-point between 110 and 220 cases, a sample with 165 cases was defined, to which a possible 10% abandonment rate was added,^12^ estimating application of 180 questionnaires (165 + 16.5 = 182). Six women refused to participate when invited. Data were not collected from five women because they were discharged early in time and did not leave the questionnaire answered. Eight did not answer all the questions. A total of 161 cases were obtained, representing a response rate of 89.45%.

## Data collection

The women were invited to participate nearly 24 h postpartum. An explanation of the theme and objectives was offered, individually, to each potential participant. Each woman who manifested initial willingness to participate was handed in a printed invitation, where the study and the data collection modality and time were presented on a specific page. The women were also informed about freedom of participation, ensuring non-prejudice for those who refused and clarifying the need for written consent. Data confidentiality and anonymization of the answers were guaranteed. The informed consent form was presented in the first contact, informing that the participant would keep one of the copies and that the other would be returned to the researcher, after being signed. The following day, approximately 48 h postpartum, the women who stated being available were asked to hand in one of the signed copies of the informed consent form to the researcher. The questionnaires were handed in to the women in opaque envelopes. The questionnaires were collected in the opaque envelopes before discharge from the ward for puerperal women. The questionnaires were applied in the first half of 2019. At the end of the questionnaire, the participant was asked if she wished to continue collaborating and her email address for a second contact, after approximately 3–4 weeks.

## Data analysis

The IBM SPSS^®^ software, version 24, was used for data analysis. A 95% confidence interval (CI) and significance level p < .05 were considered.

The analysis referred to the descriptive measures (central tendency and dispersion). For the analysis of the psychometric properties, the following was considered: (1) dimensionality analysis, (2) reliability assessment and (3) validity assessment.

## Dimensionality analysis

Dimensionality analysis was performed through the following procedures: (a) Principal Components Factor Analysis (PCFA), estimating a variance > .50% in the factors to be extracted;^12^ and (b) parallel analysis, expecting clarification between the empirical and random variance.^27^

## Reliability assessment: reliability, stability, internal consistency and equivalence

The reliability assessment was performed using the procedures presented below.

(a) Split-half test on the instrument of the first moment, expecting a value between .70 and .80, which is considered acceptable;^27^ (b) test–retest time stability, expecting a minimum value of .70;^12^ (c) Intraclass Correlation Coefficient (ICC), considering reliability < .50 as poor, values from .50 to .75 as moderate, values between .75 and .90 as good and values > .90 as excellent;^12^ (d) internal consistency or homogeneity (unacceptable < .50; questionable between .50 and .60; acceptable between .60 and.70; good between .70 and .80; very good between .80 and .90 and excellent > .90).^12^

### Validity assessment

Validity assessment resulted from (a) criterion validity, (b) construct validity, (c) structural or factor validity and (d) cross-cultural validity.

Criterion validity: (a) concurrent validity, observed through Spearman’s CEQ correlation, between the first and second evaluation moments, expecting an r_S_ coefficient > .70,^12,28^ and (b) predictive validity.

Concerning construct validity, a convergent validity was observed, expecting CEQ to be significantly correlated (p < .05; r > .400) with the ‘memory about pain intensity’ and ISPRL variables. In construct validity, discriminant validity was analysed, considering two groups of women in the CEQ scores: those who, in the ‘interaction with the midwife’ variable, recognize that such interaction has occurred versus those who deny it.^27,29–31^

When cross-cultural validity was analysed, similarities between this study and others in a similar population were explored.

### Floor and ceiling effects

The floor and ceiling effects were evaluated, setting up a cut-off point of 15%.^32^

The IBM SPSS software, version 24, was used. A significance level of .05 was considered.

## Ethical considerations

In favour of preserving intellectual property, the original author^5^ was contacted by email, requesting permission to use the instrument. The response by email was positive. According to the obituary (i.e. https://everloved.com/life-of/anna-decker/), she passed away recently. This study continued in the second half of 2019; the academic project, which, following the analysis by the Board of Directors, was deferred to the Ethics Commission of a Hospital from the South of the country (Ethics Commission of Hospital do Espírito Santo de Évora, Portugal), obtained a positive opinion (number 785).

## Results

### Sociodemographic and obstetric characteristics

The mean age of the participants was 31.05 (SD = 4.87) years, varying from 20 to 43 years. Most of them were married or lived in stable unions (n = 148; 91.9%). Regarding schooling, the most represented category is 12th grade (n = 72; 44.7%).

Most women attended eight or more prenatal consultations (n = 70; 43.5%), especially in private offices/institutions (n = 78; 48.4%). At the date of admission to the maternity ward, most of them presented 39–40 gestational weeks (n = 103; 64%). The majority did not take a birth plan to the maternity ward (n = 131; 81.4%). In the peripartum phase, 98 (60.9%) received Epidural analgesia. Nearly 70.2% (n = 113) had eutocic deliveries. For most women (n = 89; 55.3%), this newborn was their first child. The children weighed a mean of 3201 kg (SD = 0.380), with a minimum of 2080 and a maximum of 4200 kg. At the first minute, except for six (3.7%) children, all had APGAR scores greater than or equal to 7 (n = 155; 96.3%). More detailed aspects of the sociodemographic and obstetric characteristics are shown in Table 1.

**Table 1.** Sociodemographic and obstetric characteristics of the participants.

Variables	Categories	n (%)
Age (years)	20–29	65 (40.4)
	30–34	53 (32.9)
	⩽35	43 (26.7)
Marital status	Single	11 (6.8)
	Married/stable union	148 (91.9)
	Divorced	2 (1.2)
Schooling	1st cycle	4 (2.5)
	9th grade	42 (26.1)
	12th grade	72 (44.7)
	Undergraduation	43 (26.7)
Number of prenatal consultations	⩾5	28 (17.4)
	6–7	63 (39.1)
	⩽8	70 (43.5)
Pregnancy surveillance locus	State institution	62 (38.5)
	Private institution	78 (48.4)
	State and private	21 (13)
Gestational time (weeks)	37–38	30 (18.6)
	39–40	103 (64.0)
	41	28 (17.4)
Birth plan	Yes	30 (18.6)
	No	131 (81.4)
Type of delivery	Vaginal eutocic	113 (70.2)
	Vaginal instrumental	48 (29.8)
Epidural analgesia	Yes	98 (60.9)
	No	63 (39.1)
Number of children	1	89 (55.3)
	2	49 (30.4)
	>3	23 (14.3)
Total		161 (100)

Regarding the LB aspects, women have pain intensity memory with M = 73.16 (SD = 24.70), out of a maximum of 100 points. The memory of the nurses’ support in pain relief during LB presented a mean of 74.42 (SD = 27.296) and the majority (n = 129; 80.1%) reported that the nurse approached them several times asking if they felt well.

Through a multiple-answer analysis, it was observed that 135 women stated at least one ISPRL strategy (26 women did not answer this question). A total of 263 strategies were mentioned by the 135 participants who answered. The most common strategy is ‘Lying down on the side’ by 126 women, thus representing 93.3% of the total number. ‘Walking and standing supported on the companion’ appeared in second place, and ‘Leaning with the back free’ was third. However, only Strategy 3 (‘Ball balance’), Strategy 8 (‘Leaning on the support leg’) and Strategy 9 (‘Balanced support on the ball’) are listed seven and four times, respectively (Table 2).

**Table 2.** Multiple answers for ISPRL.

		Answers		% of cases
		N	%	
Multiple answers	Strategy LB1	126	47.9	93.3
	Strategy LB2	30	11.4	22.2
	Strategy LB3	7	2.7	5.2
	Strategy LB4	26	9.9	19.3
	Strategy LB5	15	5.7	11.1
	Strategy LB6	22	8.4	16.3
	Strategy LB7	29	11.0	21.5
	Strategy LB8	4	1.5	3.0
	Strategy LB9	4	1.5	3.0
Total		263	100.0	194.8

LB: Labour.

Dichotomy group tabulated at value 1.

## Analysis of CEQ

### Structural validity

The structure of CEQ was observed through PCFA. Adequacy of the sample was confirmed through the Kaiser–Meyer–Olkin test (KMO = .734; Bartlett’s Sphericity test = $\chi_{\left(231\right)}^{2}$ = 2520.217; p < .001). After introducing the 22 manifest variables, the spontaneous solution revealed six factors with eigenvalues greater than 1, in which the first explained 34.881% of the variance and the six factors, 74.158% of the variance. The screen plot suggested four factors.

PCFA was performed by applying Varimax rotation, requiring four factors, according to the slope diagram^12^ and the original model, limiting factor weight to ⩽ .40.^12^ The explained variance is presented in Table 3.

**Table 3.** Total variance explained with 22 items.

Component	Initial eigenvalues			Extraction sums of squared loadings			Rotation sums of squared loadings		
	Total	% of variance	Cumulative %	Total	% of variance	Cumulative %	Total	% of variance	Cumulative %
1	7.674	34.881	34.881	7.674	34.881	34.881	5.825	26.479	26.479
2	2.638	11.991	46.872	2.638	11.991	46.872	3.630	16.501	42.981
3	1.982	9.007	55.879	1.982	9.007	55.879	2.293	10.423	53.404
4	1.606	7.301	63.180	1.606	7.301	63.180	2.151	9.776	63.180
5	1.379	6.268	69.448						
6	1.036	4.710	74.158						
7	.893	4.057	78.215						
8	.764	3.473	81.689						
9	.622	2.829	84.518						
10	.578	2.627	87.144						
11	.474	2.156	89.301						
12	.436	1.982	91.283						
13	.384	1.745	93.028						
14	.364	1.655	94.682						
15	.251	1.140	95.823						
16	.192	.875	96.697						
17	.182	.828	97.525						
18	.168	.762	98.288						
19	.141	.639	98.927						
20	.098	.446	99.373						
21	.079	.359	99.732						
22	.059	.268	100.000						

Extraction method: principal components analysis.

**Figure 3.**
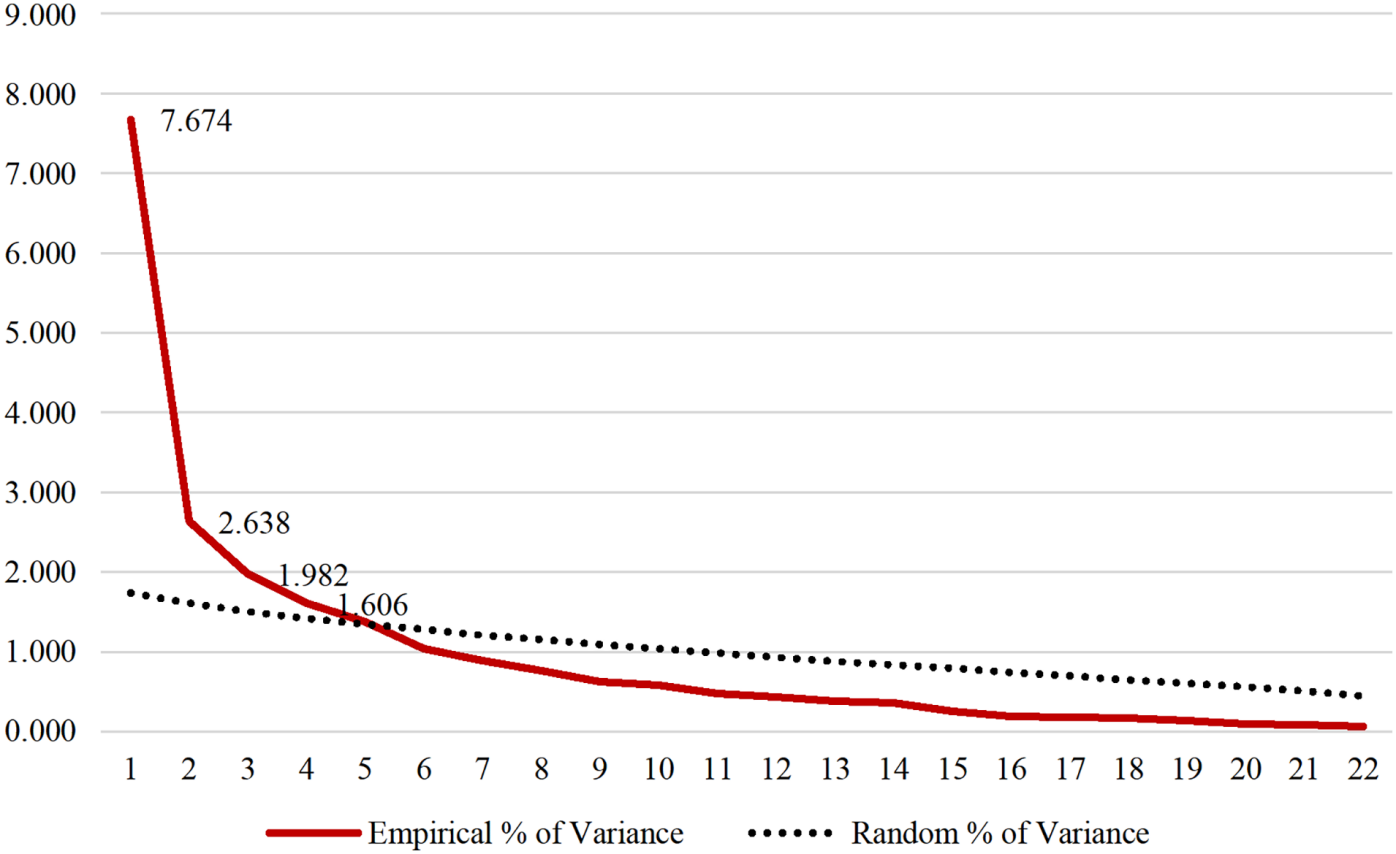
Parallel analysis of the variance of the empirical and random factors.

To clarify the dimensionality presented by the CEQ construct when adapted to the Portuguese language, parallel analysis was used, through syntax in SPSS. Thus, the behaviour of the eigenvalues corresponding to the empirical matrix was observed, given the random data eigenvalues. In the graphical representation, the construct suggests that it is four dimensional, as four factors emerge in the empirical matrix, whose magnitude of variance is higher than the random data values. Figure 3 presents the intersection of the two matrices.

The idea of four factors was maintained; however, as items 17, 18 and 22 had representation in more than one factor, it was decided to remove the one whose difference in factorial weight in both columns of values was less than .100 (Item 17: Difference in factor weight = .032) and the second PCFA was performed without Item 17, including the 21 variables.

Adequacy of the sample was maintained (KMO = .722; Bartlett’s Sphericity test = $\chi_{\left(210\right)}^{2}$ = 2203.630; p < .001). The slope diagram suggested four factors. The commonalities vary between .764 and .346 (Table 4).

**Table 4.** Commonalities loadings.

	Commonalities	
	Initial	Extraction
CEQ1	1.000	.692
CEQ2	1.000	.635
CEQ4	1.000	.423
CEQ6	1.000	.505
CEQ7	1.000	.709
CEQ10	1.000	.735
CEQ11	1.000	.764
CEQ12	1.000	.737
CEQ13	1.000	.765
CEQ14	1.000	.458
CEQ15	1.000	.759
CEQ16	1.000	.730
CEQ18	1.000	.691
CEQ19	1.000	.442
CEQ3	1.000	.531
CEQ5	1.000	.623
CEQ8	1.000	.651
CEQ9	1.000	.682
CEQ20_G	1.000	.346
CEQ21_G	1.000	.556
CEQ22_G	1.000	.693

CEQ: Childbirth Experience Questionnaire.

Extraction Method: Principal Components Analysis.

The explained variance of the first factor is 33.345%, with the set of four factors explaining 62.517% of the total variance of the measure (Table 5).

**Table 5.** Total variance explained with 21 items.

Component	Initial eigenvalues			Extraction sums of squared loadings			Rotation sums of squared loadings		
	Total	% of variance	Cumulative %	Total	% of variance	Cumulative %	Total	% of variance	Cumulative %
1	7.002	33.345	33.345	7.002	33.345	33.345	5.582	26.583	26.583
2	2.584	12.304	45.649	2.584	12.304	45.649	3.132	14.914	41.498
3	1.947	9.273	54.922	1.947	9.273	54.922	2.279	10.854	52.352
4	1.595	7.595	62.517	1.595	7.595	62.517	2.135	10.166	62.517
5	1.379	6.565	69.083						
6	1.025	4.881	73.964						
7	.892	4.248	78.212						
8	.735	3.501	81.713						
9	.622	2.960	84.673						
10	.575	2.739	87.411						
11	.468	2.227	89.639						
12	.387	1.845	91.484						
13	.374	1.780	93.264						
14	.344	1.637	94.902						
15	.245	1.167	96.068						
16	.188	.895	96.963						
17	.175	.835	97.798						
18	.158	.755	98.553						
19	.141	.670	99.222						
20	.091	.433	99.656						
21	.072	.344	100.000						

Extraction Method: Principal Components Analysis.

In PCFA, the interpretation of the four dimensions suggests the following: (a) the ‘Participation’ dimension maintains three original items (10, 11 and 12), (b) the ‘Professional Support’ dimension was reduced to four items (13, 14, 15 and 16) and (c) the ‘Own Capacity’ and ‘Perceived Safety’ dimensions evidenced a different organization of the items in this sample. Thus, observing PCFA, these dimensions were renamed to (a) ‘Own Performance’ and (b) ‘Own Threshold’. The newly named dimension ‘Own Performance’ consists of 10 items, 5 from the previous ‘Own Capacity’ dimension and 5 from the previous ‘Perceived Safety’ dimension (1, 2, 4, 6, 7, 8, 9, 18, 19 and 22, respectively). The newly named dimension ‘Own Threshold’ consists of four items (3, 5, 20, 21). The organization of the factors can be found in Table 6.

**Table 6.** Components loadings after the second factor analysis with varimax rotation.

	Component			
	1	2	3	4
CEQ1-O trabalho de parto decorreu como eu esperava The labour progress went as I had expected	.823			
CEQ9-Algumas das minhas recordações sobre o parto fazem-me sentir deprimida* Some of my memories from the labour process make me feel depressed*	−.819			
CEQ7-Tenho muitas lembranças positivas do parto I have many positive memories from the labour process	.813			
CEQ2-Senti-me forte durante o trabalho de parto e o nascimento I felt strong during labour and birth	.774			
CEQ8-Tenho muitas recordações negativas do parto* I have many negative memories from the labour process*	−.701			
CEQ18-A minha impressão sobre as competências dos profissionais de saúde fez-me sentir segura My impression of the medical competence made me feel secure	.697	.449		
CEQ22_G-Nível de segurança que senti durante o parto Experienced level of sense of security	.693	.437		
CEQ6-Senti-me feliz durante o trabalho de parto e o nascimento I felt happy during labour and birth	.660			
CEQ4-Senti capacidades durante o trabalho de parto e o nascimento I felt capable during labour and birth	.505			
CEQ19-Senti que lidei bem com a situação I felt that I handled the situation well	.503			
CEQ15-A minha parteira foi-me informando sobre o que ia acontecendo durante o trabalho de parto My midwife kept me informed about what was happening during labour		.847		
CEQ13-A minha parteira dedicou-me o tempo necessário My midwife devoted enough time to me		.827		
CEQ16-A minha parteira compreendeu as minhas necessidades My midwife understood my needs	.403	.746		
CEQ14-A minha parteira dedicou o tempo necessário ao meu marido/companheiro My midwife also devoted enough time to my partner		.535		
CEQ11-Eu senti que poderia ter decidido qual a posição do parto I felt I could choose the delivery position			.867	
CEQ10-Eu senti que poderia ter escolhido se queria estar levantada ou deitada I felt I could choose whether I should be up and moving or lie down			.807	
CEQ12-Eu senti que poderia ter escolhido as formas de aliviar a dor I felt I could choose which pain relief method to use			.778	
CEQ5-Estava cansada durante o trabalho de parto e o nascimento* I felt tired during labour and birth				−.740
CEQ3-Senti-me assustada durante o trabalho de parto e o nascimento* I felt scared during labour and birth				−.699
CEQ21_G-Nível de controlo que senti durante o parto Experienced level of control				.582
CEQ20_G-Intensidade da dor que senti no trabalho de parto* Experienced level of labour pain in dilatation stage*				−.551

CEQ: Childbirth Experience Questionnaire.

Extraction Method: Principal Components Analysis.

Rotation Method: Varimax with Kaiser Normalization.

Rotation converged in seven iterations.

Item CEQ20_G was removed, as justified in the Reliability section. *inverted items.

The items with negative formulation (3, 5, 8, 9 and 20) were reversed and the CEQ analysis continued, now considered with 21 items.

### Reliability analysis

To assess reliability of CEQ, item-total correlations, internal consistency through Cronbach’s alpha coefficient and ICC were used, considering application of the scale at the first and second moments, with 89 cases, considering the new application between 3 and 4 weeks later.

In the ‘Participation’ subscale, the item-total corrected correlations are between .572 (Item 12) and .667 (Item 10). This subscale presents Cronbach’s alpha coefficients of .782 in the first phase and of .807 in the second application. ICC was .699 (CI = .538–.801).

The ‘Professional Support’ subscale shows item-total correlations with coefficients between .661 (Item 16) and .757 (Item 15). This subscale presents a Cronbach’s alpha value of .831 in the initial application and of .782 in the second. ICC was .653 (CI = .469–.771).

In the ‘Own Performance’subscale, corrected item-total correlations between .429 (item 19) and .753 (item 8) were observed. Cronbach’s alpha coefficient was .910 in the first application and .840 in the re-test application. ICC was .641 (CI = .466–.770).

The ‘Own Threshold’ subscale presents corrected item-total correlations between .275 (Item 20) and .463 (Item 5). In internal consistency, Cronbach’s alpha was .586. In this subscale, the analysis of the set of items reveals that, by removing Item 20, Cronbach’s alpha drops to .593, which is why such decision was made. In the second application of the scale, the Cronbach’s alpha coefficient was .714. ICC was .742 (CI = .604–.829).

Without Items 17 and 20, in the total scale, it was observed that the corrected item-total correlations presented coefficients between .103 (Item 10) and .732 (Item 22_G). Cronbach’s alpha coefficient was .873 in the initial data collection and .823 in the retest. ICC was .673 (CI = .580–.788), according to Table 7.

**Table 7.** Reliability of CEQ in the test and retest.

	No. of items	Moment 1 (n = 161) Cronbach’s α	Moment 2 (n = 89) Cronbach’s α	ICC Moment 1 versus Moment 2
Own performance	10	.910	.840	.641
Professional support	4	.831	.782	.653
Participation	3	.782	.807	.699
Own threshold	3	.593	.714	.742
Total scale	20	.873	.823	.673

ICC: Intraclass Correlation Coefficient.

### Time stability and precision assessment

Also referring to reliability, time stability was analysed through Spearman’s correlation, between CEQ at the first moment and CEQ_T2 (n = 89). A positive association (r values = .659; p ⩽ .001) with statistical significance was observed.

The precision assessment of the scale was analysed using a split-half test, randomly introduced by the software. The first half resulted in an alpha value of .734 and the second half yielded a result of .802. Correlation between both halves was .786, with an alpha value of .880, by Spearman–Brown correction.

### Floor and ceiling effects

The floor and ceiling effects were observed through the sum obtained in the total scale and in the subscales, at the first and second application moments. Floor and ceiling effects of 15% were considered (Table 8).

**Table 8.** Floor and ceiling effects.

	Total scale Min 20–Max 80		Own performance Min 10–Max 40		Professional support Min 4–Max 16		Participation Min 3–Max 12		Own threshold Min 3–Max 12	
	Floor	Ceiling	Floor	Ceiling	Floor	Ceiling	Floor	Ceiling	Floor	Ceiling
Moment 1 (15%: 24 cases)	0	0	0	15 (9.3)	7 (4.3)	29 (18.0)	69 (42.9)	0	0	12 (7.5)
Moment 2: Retest (15%: 13 cases)	0	0	0	5 (5.6)	2 (2.2)	11 (12.4)	42 (47.2)	0	0	9 (10.1)

### Construct validity

In construct validity, face validity was considered, which was assumed to be reached, given that no questions were asked by the respondents. It was not possible to monitor the response time in the actual application of the questionnaire, as it was collected the day after delivery.

The analysis proceeds with non-parametric tests, as both in the total scale and in the subscales, non-normality of the distributions was verified (p < .001) through the Kolmogorov–Smirnov test with Lilliefors correction.

#### Convergent validity

The convergent validity test was performed through the correlation between the ISPRL variable and CEQ_total. Through a Spearman’s correlation, a direct association was observed between both variables (r_s_ = .209; n = 148; p = .011), meaning that more positive birth experiences are associated with the application of more positioning strategies for pain relief. Convergent validity was also tested with a measure of pain memory in LB, observing a significant inverse association (r_s_ = −.277; n = 161; p < .001),

#### Discriminant validity

**Figure 4.**
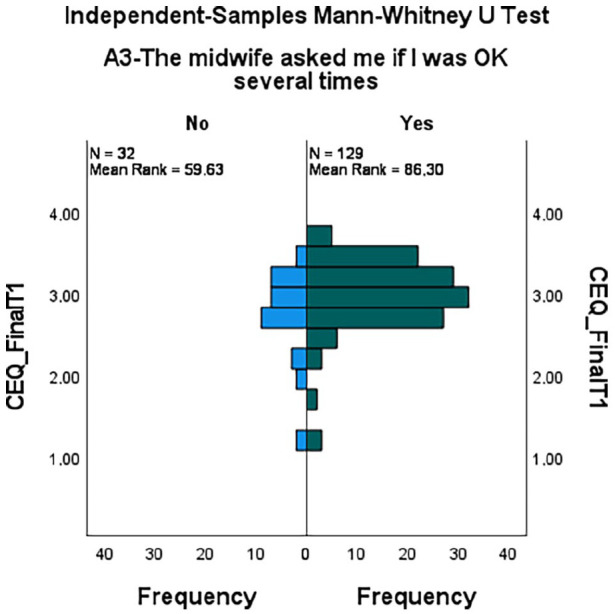
Mann–Whitney test for independent samples.

For proof of this property, a non-parametric Mann–Whitney test was performed, considering CEQ *versus* ‘Attitude of the midwife during LB: she asked several times if I was OK’. A higher mean rank was observed in the women who recognized the interaction with the midwife (n = 129; Mean Rank = 86.30) than in those who did not recognize it (n = 32; Mean Rank = 59.62), with significant differences (U = 2748.000; Z = 2.905; p = .004), as shown in Figure 4.

### Cross-cultural validity

It is observed that the ‘Participation’ and ‘Professional Support’ subscales remain approximately with the same organization of items in all four languages, while in the Own Capacity/Own Performance and Perceived Safety/Own Threshold subscales, there is exchange of some items. It is verified that, as in the case of the original instrument,^5^ the studies conducted in the United Kingdom, Spain and Slovakia^13–15^ maintain 22 items, with Cronbach’s alpha coefficients that vary between .90 and .62, although not all studies present Cronbach’s alpha coefficients on the full scale and subscales. Organization of the items coincides with the original scale, except for the study conducted in Spain, where Item 18 is moved from the Perceived Safety subscale to the Professional Support subscale. Both in the study carried out in China^16^ and in the current one, the number of items drops to 19 and 20, respectively, after moving some of them. The studies carried out in other countries with application of CEQ show results that are presented in Table 9.

**Table 9.** CEQ properties in studies carried out in other countries.

	Locus	Sample of puerperal women	Application of CEQ	Total scale		Own capacity		Professional support		Perceived safety		Participation	
				No. of items	Cronbach’s α	No. of items	Cronbach’s α	No. of items	Cronbach’s α	No. of items	Cronbach’s α	No. of items	Cronbach’s α
Dencker et al.^5^	Sweden (original)	1177 (age: 23–32 years) Primiparous women	4 weeks postpartum	22	Not available	8	.820	5	.880	6	.780	3	.620
Items						1, 2, 4, 5, 6, 19, 20, 21		13, 14, 15, 16, 17		3, 7, 8, 9, 18, 22		10, 11, 12	
Walker et al.^13^	United Kingdom	206 (age: 37–41 years) Primiparous women	1 month postpartum	22	.900	8	.790	5	.940	6	.830	3	.720
Items						1, 2, 4, 5, 6, 19, 20, 21		13, 14, 15, 16, 17		3, 7, 8, 9, 18, 22		10, 11, 12	
Soriano-Vidal et al.^14^	Spain	226 (age: 18–31 years) Primiparous and multiparous women	1–3 months postpartum	22	.880	8	.800	6	.880	5	.750	3	.680
Items						1, 2, 4, 5, 6, 19, 20, 21		13, 14, 15, 16, 17, 18		3, 7, 8, 9, 22		10, 11, 12	
Zhu et al.^16^	China	1747 (age: 18–47 years) Primiparous and multiparous women	2–3 days postpartum	19	.880	6	Not available	6	Not available	4	Not available	3	Not available
Items						1, 2, 4, 6, 7, 19		13, 14, 15, 16, 17, 18		3, 5, 8, 9		10, 11, 12	
Maskalova et al.^15^	Slovakia	161 (Age: 18–42 years) Primiparous women	2–4 days postpartum	22	Not available	8	Not available		Not available	6	Not available		Not available
Items						1, 2, 4, 5, 6, 19, 20, 21		13, 14, 15, 16, 17		3, 7, 8, 9, 18, 19		10, 11, 12	
Current study	Portugal	161 (age: 20–43 years) Primiparous and multiparous women	2 days postpartum	20	.873	9	.910	4	.831	4	.593	3	.782
Items/designation						Own performance 1, 2, 4, 6, 7, 8, 9, 18, 19, 22		original items 13, 14, 15, 16		Own threshold 3, 5, 21		original items 10, 11, 12	

CEQ: Childbirth Experience Questionnaire.

### Descriptive statistics of CEQ

Having performed the psychometric tests, the descriptive statistics of the total scale and subscales are presented since, for being ordinal variables, the items do not allow for central tendency and dispersion measures. It is observed that the dimension that is most valued by the women is ‘Own Performance’, followed by ‘Professional Support’ (Table 10).

**Table 10.** Descriptive statistics of the CEQ version under study.

		M (SD)	Min	Max	Median
Own performance	CEQ1	3.321 (0.6256)	1.20	4.00	3500
	CEQ9*				
	CEQ7				
	CEQ2				
	CEQ8*				
	CEQ18				
	CEQ22_G				
	CEQ6				
	CEQ4				
	CEQ19				
Professional support	CEQ15	3.059 (0.842)	1.00	4.00	3250
	CEQ13				
	CEQ16				
	CEQ14				
Participation	CEQ11	1.650 (0.710)	1.00	3.67	1.660
	CEQ10				
	CEQ12				
Own threshold	CEQ5*	2.56 (0.718)	1.33	4.00	2333
	CEQ3*				
	CEQ21_G				
Total scale	20 items	2.90 (0.489)	1.20	3.80	3.00

CEQ: Childbirth Experience Questionnaire.

* inverted items.

## Discussion

The designation attributed to the design of this study can be controversial, the reason why a reference in the discussion will be interesting. Most validation studies are designated as cross-sectional, as the approach to the participants presents no continuity beyond data collection for the retest. These studies are also designated as methodological, although less frequently. In a review of several studies, it is verified that designation regarding the design of validation studies lies in a grey zone, perhaps constituting a separate and different entity from other cross-sectional studies.^33^

## Sociodemographic and obstetric characteristics

The sample has characteristics that approximate the study by the original author and those carried out in other geographical spaces, both in Europe and in Asia,^5,13–16,34^ suggesting that the current validation into European Portuguese is appropriate. CEQ availability in several languages is important, as it allows performing multicentre studies.

Intercultural equivalence through translation and back-translation processes was a requirement, given the linguistic–cultural constraints, when using different words for the same idea. Although English proficiency in the Portuguese population is high (e.g. seventh place in the EF EPI 2021 report),^35^ the choice to apply CEQ both in the Portuguese and English languages to each participant, as ideally recommended in the literature,^12^ was not included. As the participants were in a transitional phase, given the puerperium period, it was feared that, due to the need for more time for interpretation and completion, presentation in both languages would lead to refusal of the invitation to participate. The face-to-face invitations, on a case-by-case basis, and the self-response in pencil–paper format, may have contributed to the current response rate, which was satisfactory when compared to online questionnaires.^36,37^

Discussions about sample size are often found in psychometric studies where Cronbach’s alpha and PCFA are frequently evaluated. According to the criterion set forth by Yurdugül (2008), a minimum of 30 cases would be sufficient to evaluate Cronbach’s alpha reliability. However, 30 cases would also be suitable for PCFA, as the largest eigenvalue was greater than .600. Conroy (2018) also considers 30 cases as sufficient.^38^ On the contrary, authors from the 1980s and 1990s (e.g. Kline, 1986; Nunnally & Bernstein, 1994) suggested a minimum of 300 cases for reliability.^39^ An adequate sample size is important, avoiding lack of test power due to underestimation or, on the contrary, unproductive effort due to overestimation. As the sample decisions were made in advance and there was no consensus,^11,38^ in the recruitment phase, it was decided to follow some authors’ guidelines for validation studies, that is, a minimum of 5 cases/item of the scale, or a minimum of 5–7 cases/item of the scale or 5–10 cases/item of the scale.^28,31,40,41^

## Dimensionality analysis

### Structural or factor validity

Structural or factor validity was observed through the PCFA, having previously guaranteed adequacy of the sample through a KMO value greater than .500,^27^ performing Varimax rotation. Choice for this rotation arises from the multidimensional character of CEQ, as the orthogonal solution maximized the high correlations and minimized the low ones.^12^

The observed variance is greater than 50% according to the previously formulated expectation.^12^ It would have been ideal to have reached 75% but 63.180% variance is sufficient.^27^ The current results are higher than the 54% of the original study^5^ than the 49.9% found in the Chinese study.^16^

A conservative attitude was chosen in renaming the factors, as there is a previous process for constructing the instrument that must be respected, despite the adequacy that must occur in validation for another culture. Naming of the dimensions was inspired by the fundamental idea of the items, close to the names given by the original author.^5^ The ‘Own Performance’ dimension reveals the perception of the woman, who, recalling the experience, self-assesses the level with which she expressed her skills. A positive self-assessment, given the difficulty of LB, empowers women. In the pregnancy–puerperium period, the biopsychosocial ritual of the female body is the exponent of femininity for some women, the integration of the woman–mother role^6^ and the realization of gender performance. The ‘Own Threshold’ dimension shows the physical vulnerability to which women in LB are subjected, recognized since immemorial times.^12^ Other reasons generate vulnerability, such as lack of control, fright and exhaustion, being related to prejudiced and non-individualized care, with maladjusted interactions with the caregivers, or even merely luminosity of the room, lack of privacy, noise or forced stimulation.^4,42^

The ‘Participation’ dimension highlights the women’s experience as central in the childbirth phenomenon and ‘Professional Support’ recognizes innate humanity, providing the best chances of life, replicating the idea of ‘obligate midwifery’ that defines us as a species.^12^ Although they are individualized dimensions in structural validity, they are complementary in assistance, contributing to good management of the ‘birth territory’. In fact, women experience the birth phenomenon supported by a guardian figure (midwife professional), who, in the care practice, has the possibility of promoting maternal satisfaction/dissatisfaction and better postpartum adaptation.^12^ The results show a similar organization of the items in these two dimensions, both in the original study and in subsequent ones.^5,13,14^

## Parallel analysis

Dimensionality is not always proven in validation studies through parallel analysis. However, it can minimize incorrect identification of factors, due to sampling error. The procedure allows PCFA to identify the number of factors in advance.^12^ The procedure was introduced in the sense of greater rigour, confirming a four-dimensional construct.

## Reliability

### Time and split-half stability

Accuracy of the overall CEQ instrument, assessed through the test–retest, showed a significant association, with a satisfactory correlation coefficient. In fact, it is close to .70, which is understood as a reliable coefficient, presenting little variation between the first and second application.^28^

The split-half test with Spearman–Brown correction, with random ordering of the items that reduces the effect of the position,^12^ showed satisfactory Cronbach’s alpha values. Divergence of the answers, both in the test–retest and in the split-half test, is understandable due to the participants’ memory bias, over which the researcher has no control. Such bias is frequently present in studies that deal with perceptions and attitudes.^12^

### ICC

The ICC values between the first and second application were moderate in the total scale and in three subscales. In the ‘Own Threshold’ subscale, ICC is already considered high, as it is above .70. ICC is an adequate reliability parameter for continuous measures^32,43^ and, in the current case, time stability has been proven. ICC is not always evaluated, but the verification contributes rigour to the research.^32^

### Internal consistency or homogeneity

In psychometric studies, measure reliability is frequently expressed through the alpha coefficient, and with low samples, the coefficients can become unstable. An important issue in psychometric studies is measure accuracy, which is frequently quantified by the reliability coefficients. The alpha coefficient developed by Cronbach (1951) is the index most commonly used to estimate the reliability of measuring instruments (Raykov, 1997) in the fields of Psychology, Education, Statistics, Sociology, Medicine, Counselling, Nursing, Political Science and Economics.

The results corresponding to internal consistency of the Own Performance reorganized subscale compete for what is observed in the subscale called ‘Own Capacity’ in the original and Spanish versions.^5,14^ The fact that it was organized in 10 items, through PCFA, will have contributed to that. In fact, the values of the Cronbach’s alpha coefficients are influenced by the number of manifest variables. A higher number of items in the domain lead to better results in internal consistency^12^

In the Own Threshold subscale, despite item-total corrected correlations above .300, the minimum acceptable^27^ showed a very low Cronbach’s alpha value. Such value is slightly above .500, considered questionable.^44^ In fact, in subscales with few items, the measure reliability analysis may present unstable coefficients. However, it is a psychological construct, in which, due to diversity, it can be understood that alpha values are lower.^27^ Diversity of experiences can be significant and, if for some women, motherhood is assumed as empowerment in gender, others will be vulnerable, perhaps already initiating the motherhood blues phase, typical of postpartum, in which women waver over their limits.

Internal consistency of the ‘Participation’ subscale presents higher Cronbach’s alpha coefficient values than the original, the English and the Spanish studies.^5,13,14^ The subscale suggests that it is consolidated in the several languages.

In the ‘Professional Support’ subscale, except for one item,^17^ the organization is similar to the original scale. The item-total correlations are satisfactory, as is the Cronbach’s alpha coefficient. This coefficient is satisfactory.^27^ In fact, if Cronbach’s alpha is low, lack of correlation between the items is assumed. However, if it is very high, it indicates high correlations between the manifest items, that is to say, redundancy.^32^

Briefly, the results suggest that, although the number of items has decreased, agreement between the items presented by Cronbach’s alpha coefficient is adequate.^12^ In the item-total correlations, the values were satisfactory and confirmed measurement of the same construct.

### Construct validity

#### Convergent validity

In convergent validity, a positive association was observed between ISPRL and CEQ_total, meaning that more satisfactory birth experiences are associated with greater use of pain relief strategies. However, the most unsatisfactory LB experiences were associated with more intense pain memories. The significant correlations, although with low coefficients, suggest guarantee of this property. A positive association of .400 was expected,^27^ which presented a lower coefficient, although significant. In fact, pain level can influence satisfaction with LB.^6^ However, memory in early postpartum is one of the neurobiological changes experienced by women,^45^ which may have influenced the answers and, thus, the correlation strength between the variables.

#### Discriminant validity

In discriminant validity, it was found that the women who remember interactions with the midwives have more satisfactory childbirth experiences. Taking the idea of the parturient women’s vulnerability, the result will be credible, validating the distinction observed between both groups of women. LB should occur centred on the woman,^19^ but this becomes more or less feasible, depending on how the ‘birth territory’ is managed.^8,9^ In fact, by dominating the professional territory, midwives have the possibility of making LB less/more medicalized, of facilitating/obstructing the maternal bond, and of valuing the physiological ability to give birth while maintaining integrity, privacy and safety.^10^

#### Cross-cultural validity

Cross-cultural validity is a parameter that evidences similarities/differences which occur between this study and others conducted with similar populations. Given that the perspective is rooted in the culture, perhaps among the published studies that used CEQ, the one carried out in Valencia is the closest in geographical terms. However, the CEQ-E data were collected 1–3 months postpartum, which makes the participants’ recall more distant when compared to the current results.^45^ Organization of the items in the ‘Participation’ subscale is the same in the several languages, suggesting consolidation in the relationships between the items. Among the current versions of CEQ, the one that suggests to be more approximate is the one validated in Slovakia,^15^ coinciding with the fact that data collection also occurred before the end of the first puerperal week. Validation of scales in the postpartum can present difficulties since, at this phase of life, psychosocial stress is high and can moderate cognitive changes, such as memory deficits.^12^ In addition to the time that elapses between delivery and application of the instrument, the emergence of postpartum or motherhood blues can also lead to typical emotional instability and affect self-disclosure of experiences.

### Construct validity

However, the results contribute to an assertion that recalls a text from the 1980s (Zeller and Carmines, 1980) where it is stated that construct validity is not defined in a single study because, on the contrary, it requires consistent results that can be found by different authors over a period of time.^46^

### Floor and ceiling effects

For the floor and ceiling effects, a 15% proportion was adopted, given that it is a value present in the quality criteria, when validating instruments in the health field.^32^ This value is not consensual, as other authors quantify the proportion in a 15%–20% range.^47^ At both moments, in the total scale and in the ‘Own Performance’ and ‘Own Threshold’ subscales, the percentage of participants who obtained the highest and lowest scores is below the cut-off point of 15%. The results indicate that, in these dimensions, the instrument is sensitive enough to detect differences in the birth experience between the participants who are at the extremes, that is, with the best and worst scores. However, in the ‘Professional Support’ subscale, the ceiling effect was present in the first application of the instrument (n = 161), surpassing the cut-off point by 3% (n = 5 cases). In the ‘Participation’ subscale, the floor effect is accentuated and was present in the test and retest, showing that, in this dimension, CEQ may not identify differences at the ends of the measuring scale. As far as it was possible to observe, this parameter is not considered in previous CEQ validation procedures. However, the presence of these effects is suggestive of the lack of extreme items and thus indicates some limitations in content validity, reducing reliability.^32^ However, the set of items in the ‘Participation’ dimension falls within a care sphere that is currently in transition. In fact, more conventional care practice models understand care during LB as keeping the woman bedridden, valuing passivity, that is, not allowing ‘Participation’ to become a reality. Currently in Portugal, recent guidelines^12^ supported by international recommendations^19^ seek to confer greater prominence to parturient women. In addition to this comment, the possible bias of the convenience sample cannot be ignored, which is related to care styles offered by the midwives to these participants or to the consequences of some forms of epidural analgesia.

### Descriptive statistics of CEQ

Despite the limitations identified in the psychometric analysis, the descriptive statistics of CEQ suggest that the instrument is adequate to describe the women’s experiences in LB. It is important that the two dimensions most valued by the participants are ‘Own Performance’ and ‘Professional Support’, with the emerging idea that assistance in LB occurs in partnership, in the beneficiary/caregiver relationship. Interpretation of the current data contributes to studies that reveal emotional and bio-constitutional aspects of human females giving birth. Regarding the emotional aspects, it is worth citing a number of authors^48^ who recognize parturients as women who are living ‘private lives in public places’, requiring allies to take care of them at a high vulnerability moment. In the bio-constitutional aspects, a retrieval of millions of years maintains the legacy of bipedalism. Specified in humans, among mammals, this legacy requires help for successful childbirth.^12^ In fact, throughout LB, women change their mood, have expectations regarding their performance and the support provided by the professionals,^5,49^ and live a transition that can empower them,^6^ although presenting care needs.^12^

The ‘Own Threshold’ dimensions are in the third appreciation position. This may contribute to self-confrontation between the LB demands and own potentialities. In fact, there is always an unknown facet in each delivery because, even if a woman is multiparous, it will always be a new delivery. This is still the belief.

‘Participation’ is the least-valued dimension and is perhaps part of the medical–technological model that currently enjoys significant adherence in healthcare; faced with the overload of medical equipment and exclusion of physiological models recommended by the WHO.^19,50^ Medicalization of childbirth can generate in parturients the expectation that delivery is something that happens in their body, for which there are medical solutions that remove pain, evaluate parameters, maintain safety and monitor the foetus, offering the possibility for them to be able to alienate themselves and become self-spectators. In fact, the WHO guidelines emphasize the importance of not overlapping the equipment potentialities to the detriment of human assistance, stimulating one-to-one care, recommending in normal LB that continuous monitoring is not used in the cardio-foetal record and that the beneficiary–user interaction occurs systematically, following LB evolution and using a Partogram.^19^ It is useful to have professional standards/guidelines to assist in childbirth, although a strict culture may not meet the parturients’ needs. Maintaining indisputable rules, not recognizing women’s individual vulnerability, devaluing cultural identity and not respecting the ‘Golden Hour’ in favour of standardized but postponable tasks, harms the parturients’ experience.^4,51^ The absence of physical and psychospiritual comfort during LB, and poor individualized care are associated with post-traumatic stress disorder.^52^

## Limitations

The convenience sample introduces limitations to the study, as only participants with easy access for the researchers were eligible. Another limitation is related to the memory bias because, although the data were collected at a moment close to delivery, recall of the event is not totally accurate. Application of the questionnaire on the second postpartum day may have been too early an option to recall the experience. Given that, in the subscales, the Cronbach’s alpha coefficient presents values between excellent and questionable, introduction of a social desirability measure could have been useful. Also, confirmatory factor analysis (CFA) and exploratory factor analysis (EFA) was not performed, and future studies need to address this issue.

## Contributions to research

This study opens perspectives for continuity of research on the topic. It will be appropriate to test more properties, such as criterion validity, in addition to performing confirmatory analyses. This study may contribute added value for future validation in African (Angola, Mozambique, Guinea-Bissau, Cape Green) or Asian (East Timor) countries where the official language is Portuguese.

## Conclusion

The analysis performed suggests that, through the current validation procedure, CEQ is a reliable and valid measure that can be applied in European Portuguese. The validation process involved reorganization of two subscales and removal of two items. The results show satisfactory psychometric properties. The scale is organized into four dimensions, with a total of 20 items. Internal consistency is satisfactory. Convergent and discriminant validity has been proven. Cross-cultural validity was synthesized based on data from other languages. Time stability was satisfactory. Through the analysis of the floor and ceiling effects, it was found that, in the ‘Participation’ subscale, there is low capacity to distinguish between respondents from the lower extremes. In the ‘Professional Support’ subscale, the ceiling effect suggests poor distinguishing ability, but in the upper extremes.

It will be useful to further investigate CEQ with samples from other puerperal contexts and to reassess its reliability. It is appropriate to test more properties, such as criterion validity. It will be useful to carry out studies in larger, random samples, covering women from other parts of the country and in other Portuguese-speaking obstetric contexts.

## Supplemental Material

sj-docx-1-whe-10.1177_17455057221128121 – Supplemental material for Childbirth Experience Questionnaire: Cross-cultural validation and psychometric evaluation for European Portuguese Click here for additional data file. Supplemental material, sj-docx-1-whe-10.1177_17455057221128121 for Childbirth Experience Questionnaire: Cross-cultural validation and psychometric evaluation for European Portuguese by Maria João Pimenta Marques, Otília Zangão, Luis Miranda and Margarida Sim-Sim in Women’s Health

sj-docx-2-whe-10.1177_17455057221128121 – Supplemental material for Childbirth Experience Questionnaire: Cross-cultural validation and psychometric evaluation for European Portuguese Click here for additional data file. Supplemental material, sj-docx-2-whe-10.1177_17455057221128121 for Childbirth Experience Questionnaire: Cross-cultural validation and psychometric evaluation for European Portuguese by Maria João Pimenta Marques, Otília Zangão, Luis Miranda and Margarida Sim-Sim in Women’s Health

## Declarations

**Ethics approval and consent to participate:** The project, analysed by the Board of Directors, was deferred to the Ethics Commission of the Hospital do Espírito Santo de Évora, Portugal, obtaining a positive opinion (number 785). The participants provided their written informed consent to participate in this study before data collection.

**Consent for publication:** Not applicable.

**Availability of data and materials:** Data are available from the corresponding author if requested.
