# Supplementary figures and images for: Childbirth Experience Questionnaire: Cross-cultural validation and psychometric evaluation for European Portuguese
Source: Womens Health (Lond). 2022 Oct 18;18:17455057221128121. doi: 10.1177/17455057221128121 (PMC9583229; doi:10.1177/17455057221128121)

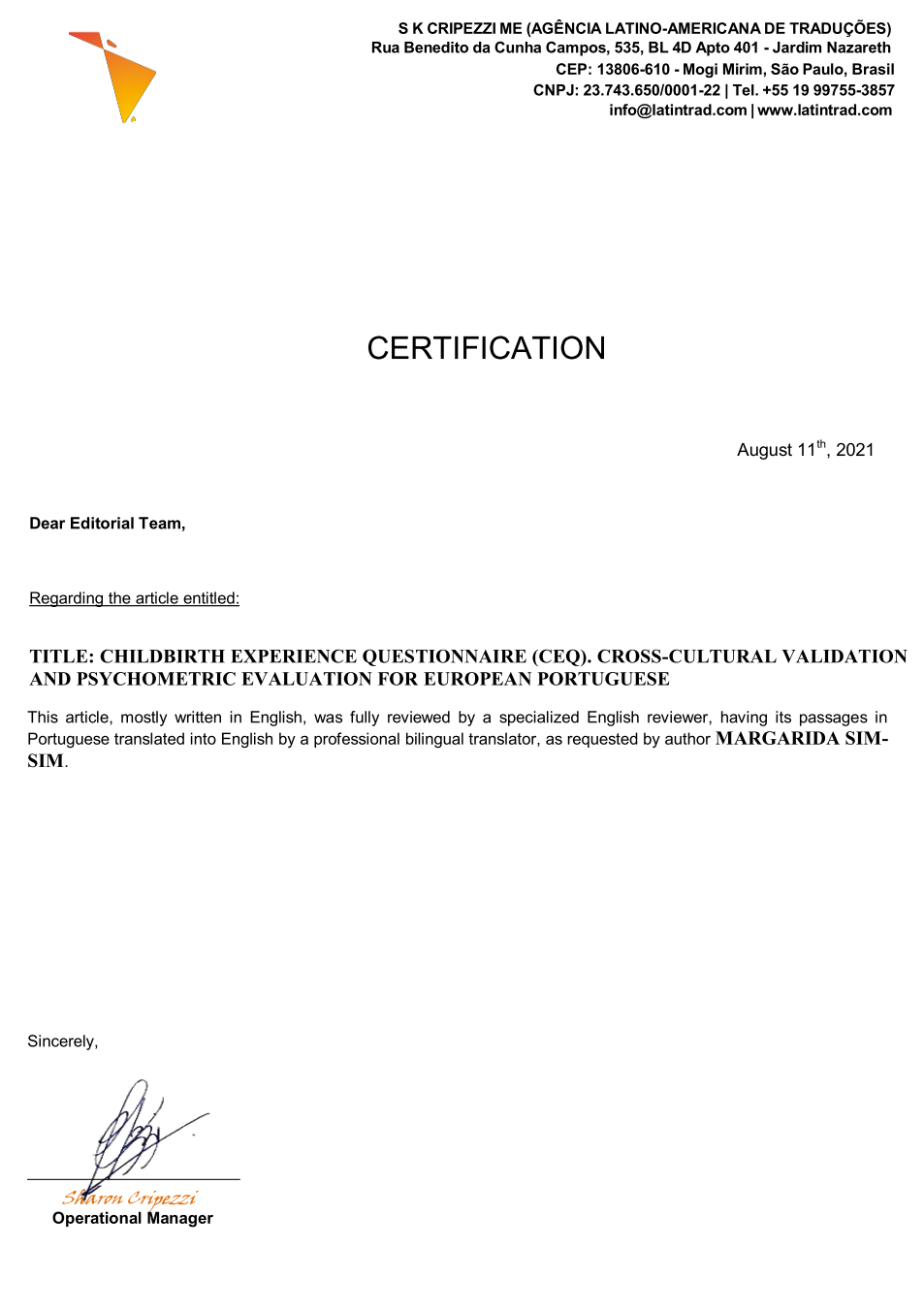

Supplement: sj-docx-1-whe-10.1177_17455057221128121 – Supplemental material for Childbirth Experience Questionnaire: Cross-cultural validation and psychometric evaluation for European Portuguese [file sj-docx-1-whe-10.1177_17455057221128121.docx]

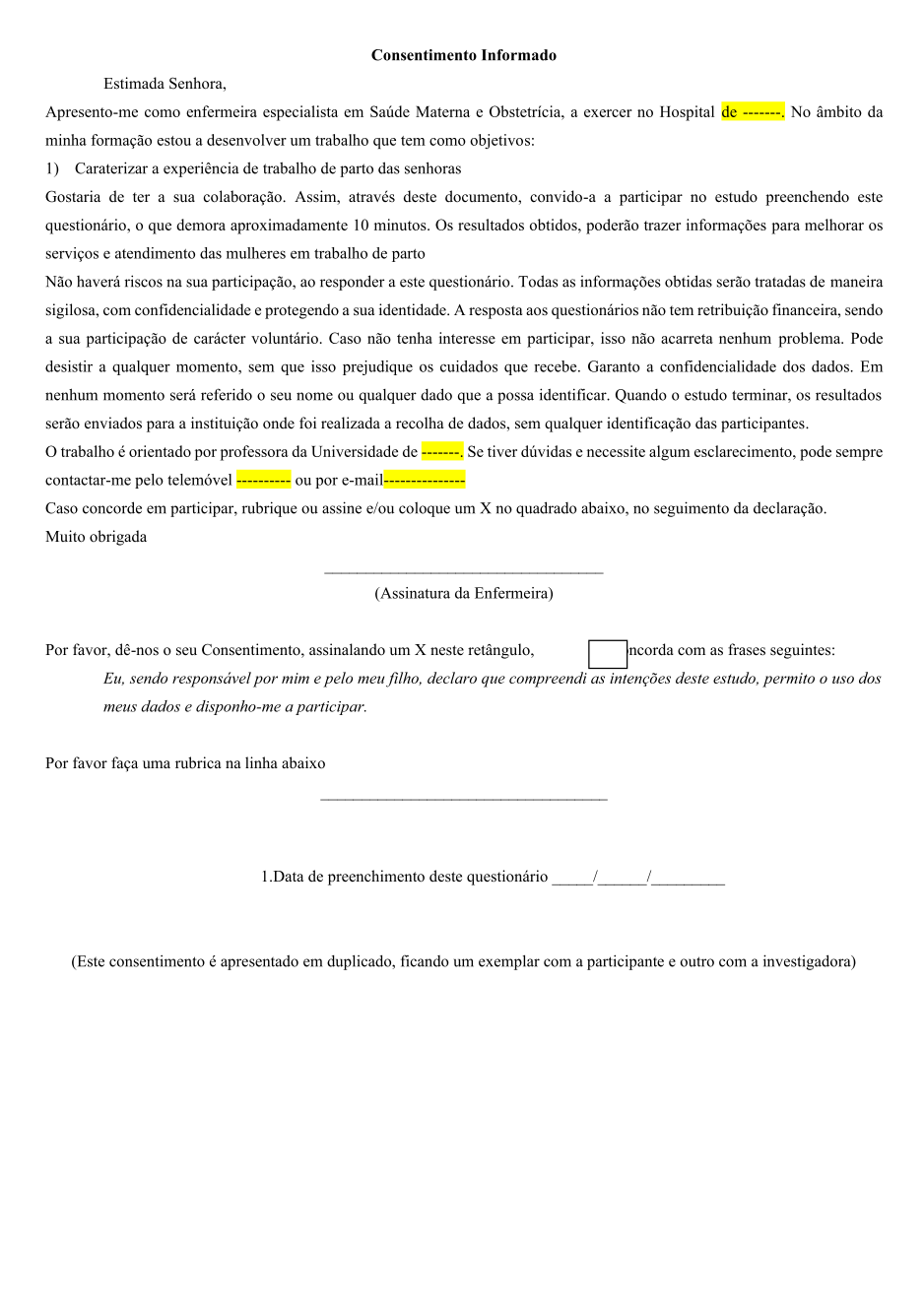

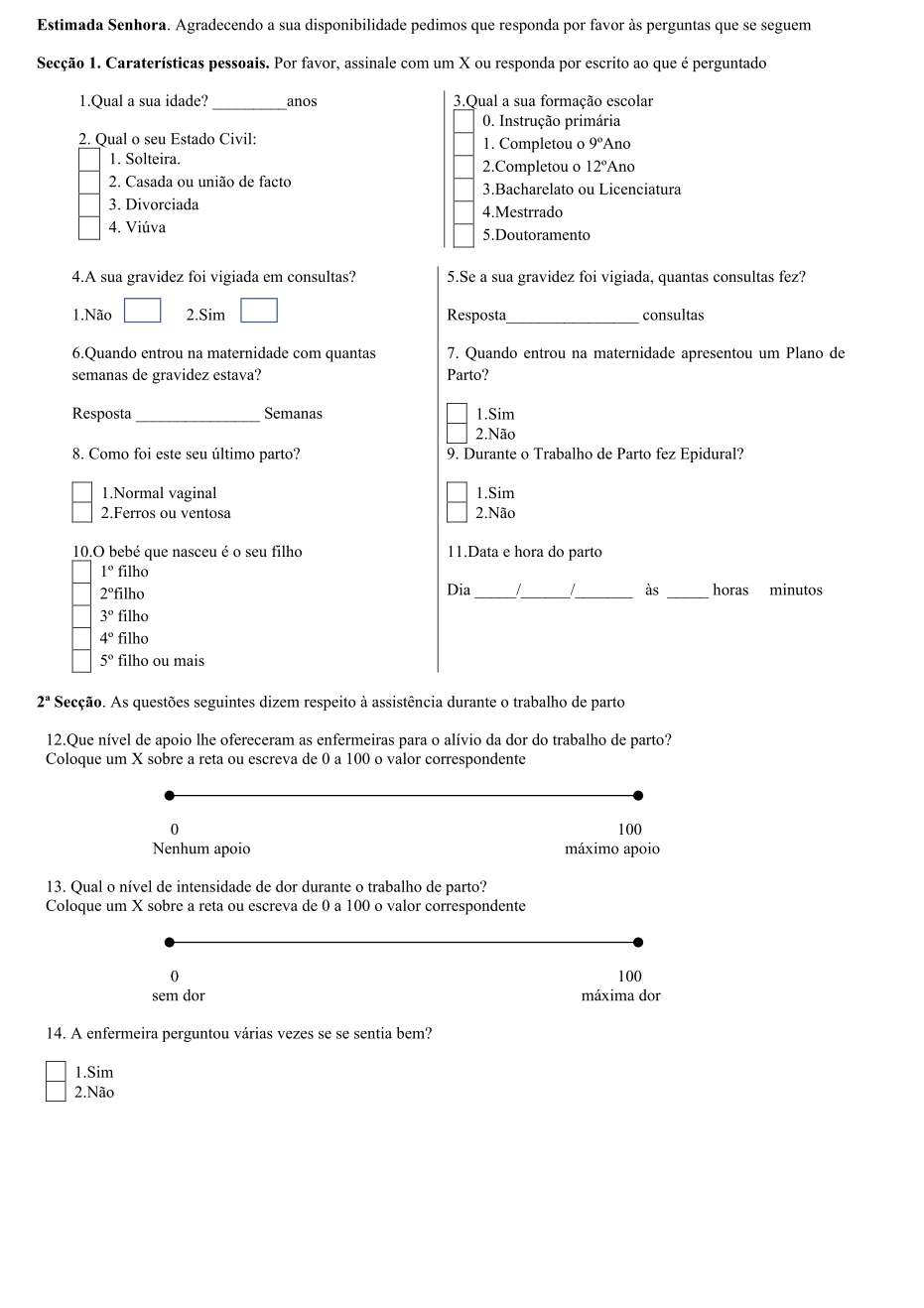

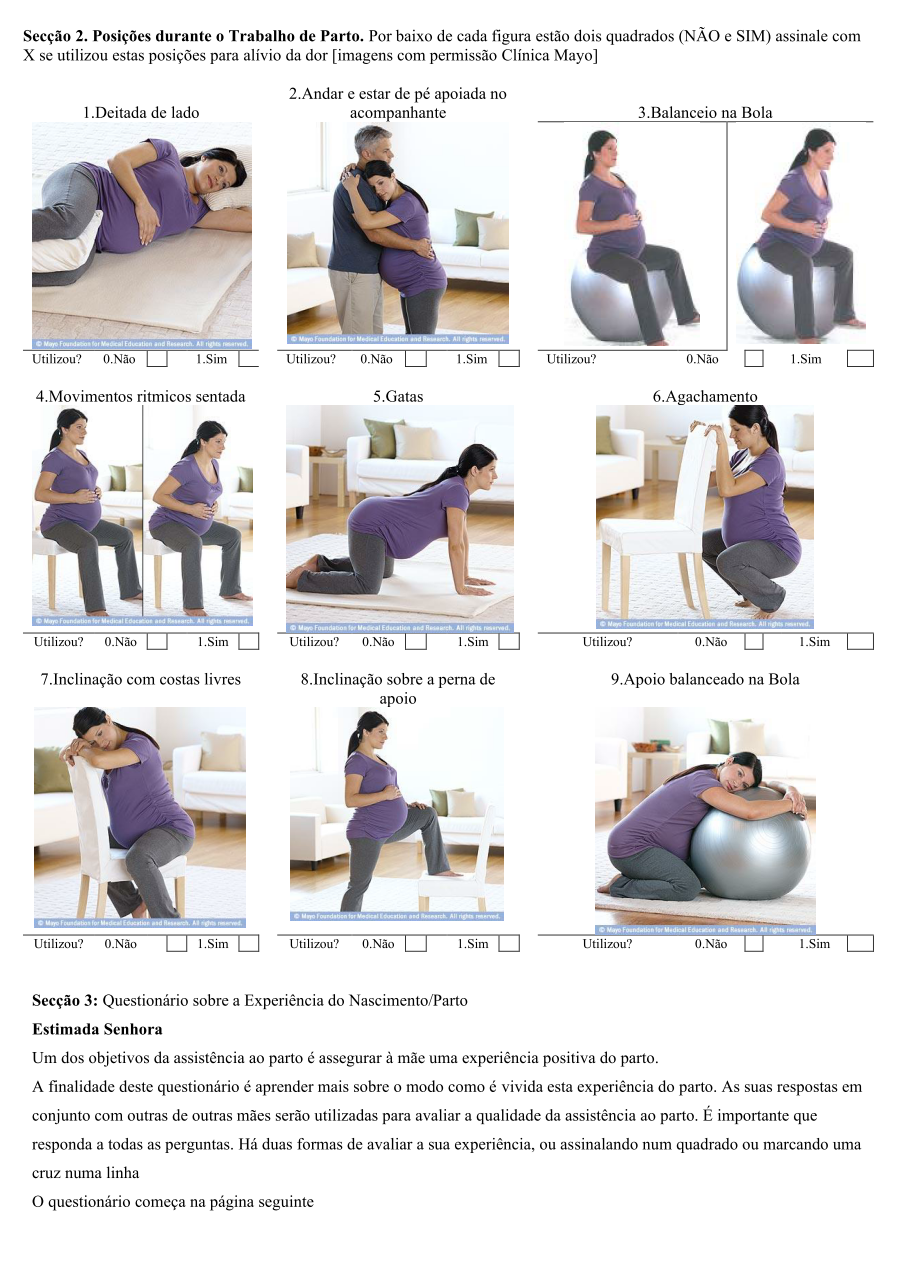

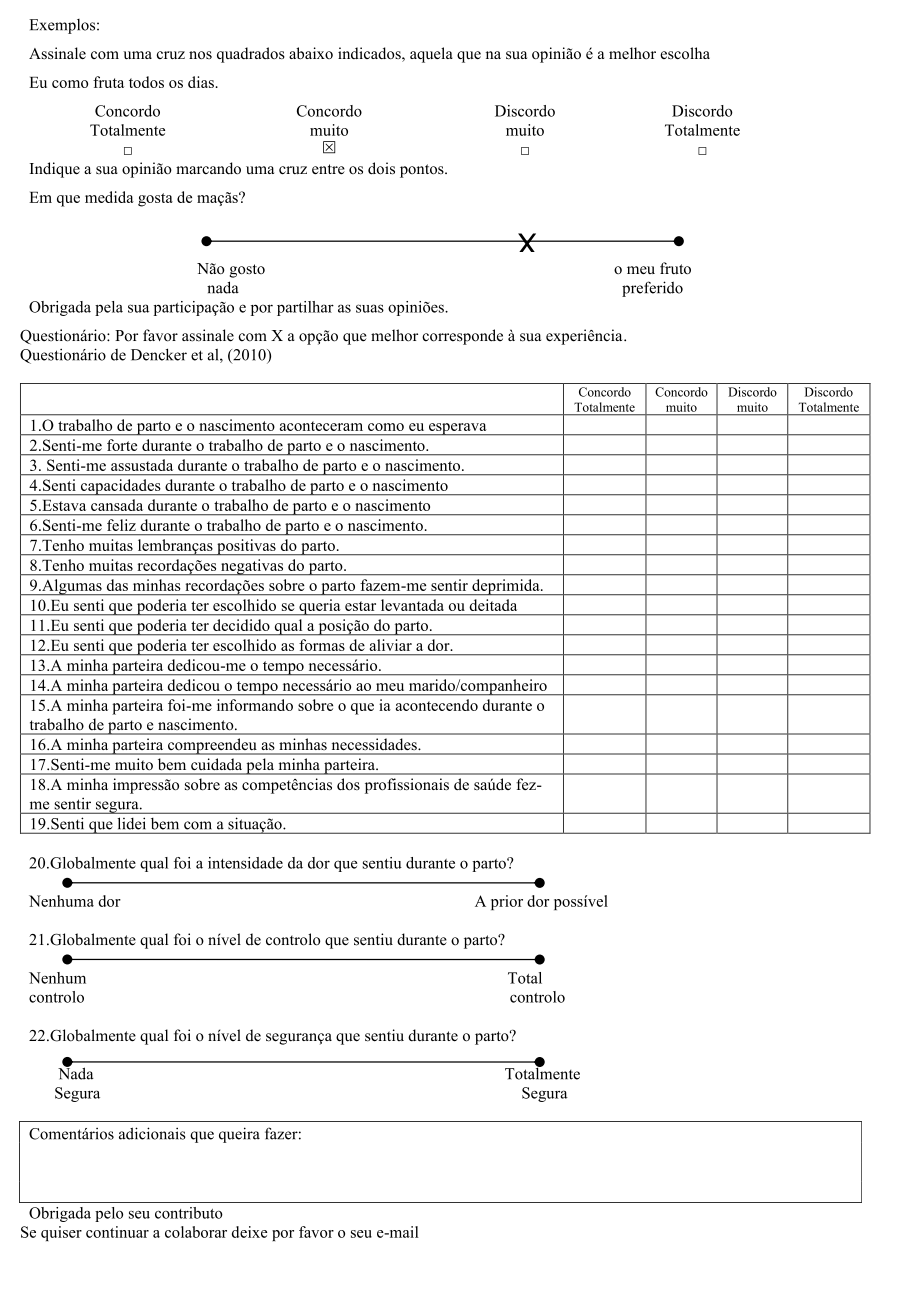

Supplement: sj-docx-2-whe-10.1177_17455057221128121 – Supplemental material for Childbirth Experience Questionnaire: Cross-cultural validation and psychometric evaluation for European Portuguese [file sj-docx-2-whe-10.1177_17455057221128121.docx]
